# Building capacity for citizen science in health promotion: a collaborative knowledge mobilisation approach

**DOI:** 10.1186/s40900-023-00451-4

**Published:** 2023-05-30

**Authors:** Samantha Rowbotham, Pippy Walker, Leah Marks, Michelle Irving, Ben J. Smith, Yvonne Laird

**Affiliations:** 1 Menzies Centre for Health Policy and Economics, School of Public Health, Faculty of Medicine and Health, The University of Sydney, Sydney, NSW Australia; 2 The Australian Prevention Partnership Centre, The Sax Institute, Sydney, NSW Australia; 3 Charles Perkins Centre, The University of Sydney, Sydney, NSW Australia; 4 Prevention Research Collaboration, School of Public Health, Faculty of Medicine and Health, The University of Sydney, Sydney, NSW Australia

**Keywords:** Citizen science, Capacity building, Knowledge mobilisation, Health promotion, Public health, Health policy

## Abstract

Policymakers and practitioners in health promotion (e.g. those working for local, state or federal government organisations or community and non-government organisations with a focus on health and wellbeing) are increasingly interested in citizen science as a means of involving the public in research and decision making. The potential benefits of citizen science approaches in health promotion include increased research capacity, incorporation of community perspectives on problems and solutions, and improved public awareness and acceptance of actions to improve health. However, health promotion practitioners and policymakers report having limited familiarity and experience with citizen science and a desire to build their capacity in these approaches. The Citizen Science in Prevention (CSP) project aims to build capacity for citizen science in health promotion by: 1) supporting the development and implementation of citizen science projects by policymakers and practitioners, 2) establishing a network of health promotion stakeholders with familiarity and interest in citizen science approaches, and 3) co-designing resources to support the use of citizen science in policy and practice contexts. A comprehensive mixed methods evaluation will establish the reach, satisfaction, and impacts that can be attributed to the capacity building intervention. This paper describes the first known initiative to build capacity in the application of citizen science approaches in health promotion and we hope that this work will assist others in the development and implementation of capacity building activities for citizen science in health promotion and beyond.

## Abstract (plain language summary)

Citizen science, the active involvement of members of the public in undertaking research, is gaining attention as a means of involving the public in research and decision making in health promotion. However, despite increasing interest in citizen science, policymakers and practitioners in health promotion (e.g. those working for local, state or federal government organisations or community and non-government organisations with a focus on health and wellbeing), lack the knowledge, skills and confidence to apply these approaches within their work. Knowledge mobilisation is a process designed to ensure research is useful for society, underpinned by researchers and non-academic partners working together to ensure that the knowledge produced is relevant and useful to those responsible for making decisions in practice. Within this paper we describe how we have used a knowledge mobilisation approach to work in partnership with health promotion agencies to develop, implement and evaluate a suite of activities aimed at building capacity in the use of citizen science approaches in health promotion.

## Background

Community engagement is a core principle of public health and health promotion [1–5], and is recognised as a key factor in ensuring that policies and programs to improve health and wellbeing are relevant to community needs and optimise limited resources [2, 16–20]. However, incorporating community perspectives into research and policymaking has proven challenging [22], and there is a recognised need to further develop capacity and infrastructure to enable health promotion researchers, policymakers and practitioners to engage with the public in meaningful ways [16, 23, 24].

Citizen science, broadly defined as “public participation and collaboration in scientific research” [1] involves members of the public (‘citizen scientists’)^1^ in a range of research activities, including developing research questions, designing project methodologies, data collection and analysis, and discussing, interpreting and disseminating research results [2–5]. A distinguishing feature of citizen science is that it brings participants into research as active contributors to the scientific process [3, 6] and involvement in citizen science projects can range from citizen scientists contributing to researcher-led projects, through to citizens developing and leading their own projects [7–11]. Citizen science has a long history in the natural sciences, particularly in counting or monitoring animals and insects, weather patterns, air and water quality, and stars and planets, and the significant contributions of citizen scientists in disciplines such as ecology and environmental science have been widely acknowledged [12–14].

### Citizen science in health promotion

Over the past decade, citizen science approaches have attracted attention in health-related disciplines [15], with a growing body of literature discussing and reporting on the application of these approaches in public health and health promotion [3, 16, 17]. The potential benefits of citizen science approaches include increased research capacity, incorporation of community perspectives on problems and solutions, and improved public awareness and acceptance of actions to improve health [3]. A recent scoping review found that citizen science approaches have been used to: identify problems from the perspective of community members; generate and prioritise solutions; develop, test and/or evaluate interventions; and/or build community capacity [16]. For example, citizen science approaches have been used to identify environmental barriers and facilitators to physical activity and healthy eating [11, 18–21], understand public perceptions of alcohol-related harm [6], and collect information to support advocacy for tobacco control policies [22].

To date, most research applying citizen science approaches in public health and health promotion has been led by academic researchers [16, 23–25]. However, there is growing interest among stakeholders within policy and practice organisations responsible for health and wellbeing (e.g., government and non-government health promotion agencies, local health districts, and local councils) in applying these approaches within their work [6, 22, 26–29]. This growing interest presents a window of opportunity to build familiarity and skills in the application of citizen science approaches and explore the value that this can add to policymaking and program development. While progress has been made to build capacity in citizen science at individual and organisational levels in Australia and internationally [30], there has been little attention to the needs of policymakers and practitioners in health promotion and public health. Given the sensitivities involved in undertaking health-related research with the public, including balancing risks and benefits to community members, collecting and protecting personal data, working with vulnerable groups, and navigating ethical review committees [31–34], there is a need to identify and address the capacity building needs of these stakeholders.

### Capacity building and knowledge mobilisation

Capacity building can be defined as “an approach to the development of sustainable skills, organisational structures, resources and commitment to health improvement in health and other sectors”, that can be undertaken with individuals, teams, organisations, and/or communities [35]. While many frameworks for capacity building exist, these often share key principles and elements such as stakeholder engagement, identifying and leveraging pre-existing capacity, building partnerships and trust, providing tailored resources and support, and developing skills and confidence [30, 36–38].

An ongoing focus of capacity building in the public health field is improving the application of evidence into policy and practice. Based on years of effort to facilitate ‘research translation’, there is growing recognition that evidence that is co-produced with knowledge users (e.g. policymakers and practitioners in public health) is more likely to be embedded in policy and practice [39, 40]. ‘Knowledge mobilisation’ is an approach to working in partnership to strengthen capacity to conduct, share and use research effectively [41] and is particularly well suited to citizen science capacity building because it is about bringing together key stakeholders to collaboratively identify solutions that address shared priorities.

Within this paper we outline our approach to implementing and evaluating a knowledge mobilisation initiative to build capacity in the use of citizen science approaches by policy and practice stakeholders in Australia. The aim of this paper is to outline the theoretical foundation to this approach coupled with a description of collaborative methods, project activities and the evaluation framework to assess the delivery and impact of capacity building efforts.

### Overview of the Citizen Science in Prevention project

The Citizen Science in Prevention (CSP) project [42] is a national collaborative initiative which seeks to achieve the following key objectives:

- Produce new knowledge on the feasibility, impacts and potential limitations of citizen science approaches within policy and practice contexts;
- Increase familiarity and acceptance of citizen science and develop knowledge, confidence and skills in the application of these approaches among policy and practice stakeholders;
- Establish a network of stakeholders with an interest in citizen science to enable sharing of experiences and insights.

It is important to note that the intention of this project was not to work directly with community members, but to enhance the skills and capacity of policymakers and practitioners to do so within their own projects. Within this work we therefore describe a suite of capacity building activities that were co-designed with policymakers and practitioners in health promotion to equip them with the knowledge, skills, and confidence to engage community members using citizen science. A glossary of terms can be found in Table 1.

**Table 1:** Glossary of terms

Term	Description
Citizen science	Citizen science enables members of the public (‘citizen scientists’) to actively contribute to the scientific research process. Citizen scientists may be involved in a range of research activities, including developing research questions, designing project methodologies, data collection and analysis, and discussing, interpreting and disseminating research results
Health promotion	Health promotion is the process of enabling people to increase control over, and to improve their health [43]. This is achieved by increasing control over the determinants of health (e.g., factors related to the social and economic environment, physical environment and the characteristics and behaviours of individuals). There are five priority action areas for health promotion as outlined in the Ottawa Charter [43]. These include to build healthy public policy; create supportive environments for health; strengthen community action for health; develop personal skills; and re-orient health services
Policy and practice stakeholders	Policy and practice stakeholders are those who work within organisations responsible for health and wellbeing (e.g., health promotion agencies, local councils, and local health districts). This may include policymakers and practitioners in health promotion and public health involved in decisions regarding the funding, design, implementation, and/or evaluation of public health policies, programs and services
Knowledge mobilisation	Knowledge mobilisation involves the co-production of research between academic and non-academic partners with the aim to ensure research evidence is both relevant and useful for society and those making evidence informed decisions in practice. As described by Phipps et al. [44] “Knowledge mobilisation helps make academic research accessible to non-academic audiences and supports collaborations between academic researchers and non-academic partners such as community-based organisations”
Capacity building	Capacity building in health promotion has been defined by the World Health Organisation (WHO) as “the development of knowledge, skills, commitment, partnerships, structures, systems and leadership to enable effective health promotion actions” [43]. Capacity building interventions are actions to improve health through the advancement of knowledge and skills among policy and practice stakeholders, expansion of organisational support and infrastructure, and the fostering of partnerships for community health [43, 45]

## Methods

### Phase 1: Application of a knowledge mobilisation approach to capacity building

**Fig. 1:**
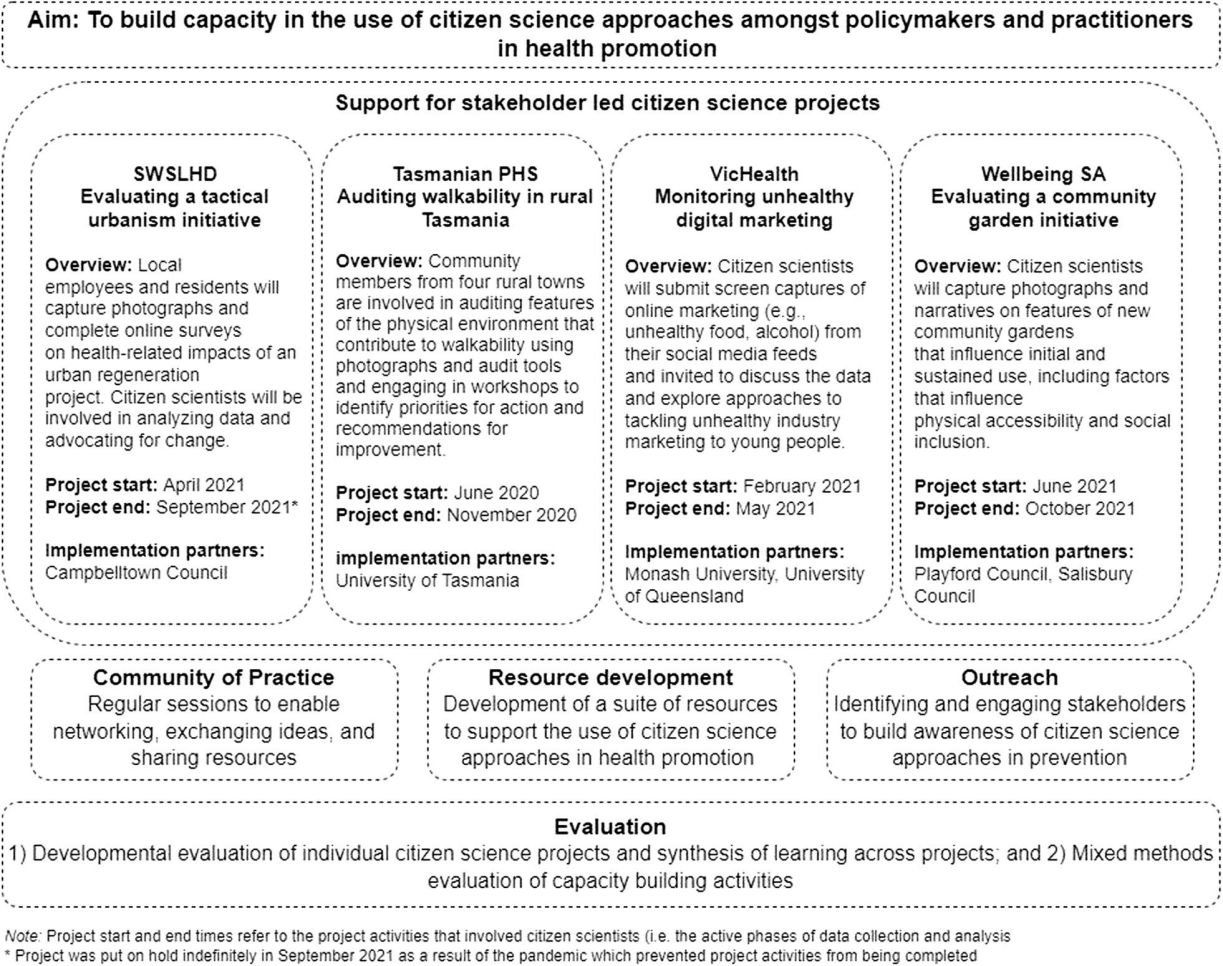
Overview of the Citizen Science in Prevention project

In line with knowledge mobilisation principles [41, 46], this project is underpinned by a commitment to co-production and working in partnership with policy and practice stakeholders at all stages of the project. We have partnered with four Australian health agencies: VicHealth, Tasmanian Department of Health, Wellbeing SA and the South Western Sydney Local Health District (SWSLHD), and representatives from each of these organisations are named investigators on the research team. Our partners play an active role in shaping this program of work, with regular opportunities to contribute to and discuss activities so that the workplan can evolve and respond to emerging needs. In developing our approach, we drew upon input from a variety of key stakeholders, recognising the expertise they bring from their own contexts. This included early discussions with our project partners to identify activities, resources and support required, and interviews and informal discussions with other health promotion stakeholders (including those working in local health districts and local councils with a role in health and wellbeing and/or community engagement). Through this process we identified several capacity building needs related to the use of citizen science, including: accessible and informative resources to assist partners in planning citizen science projects and talking about these approaches with other key stakeholders; opportunities to share insights, experiences, and challenges with others who are using these approaches; advice and support from experienced citizen science researchers/practitioners in developing projects; and evidence and examples to demonstrate the feasibility and impacts of citizen science approaches in different policy and practice contexts. The project is being implemented over three years and consists of several interrelated components which aim to address the capacity building needs identified above, namely: (1) supporting the development and implementation of four stakeholder-led citizen science projects (see Fig. 1) [47]; (2) developing resources to support the use of citizen science by policy and practice stakeholders; (3) facilitating a Community of Practice (CoP) to bring together interested stakeholders to share information and insights, and (4) outreach activities to establish a wider network of stakeholders with familiarity and interest in citizen science approaches.

As noted above, knowledge mobilisation approaches are particularly suited to working in partnership to strengthen capacity to conduct, share and use research effectively. Within this program of work, we drew on knowledge mobilisation domains established by Davies, Powell and Nutley [48]. These include: ‘researching in practice’ to provide hands-on support for stakeholder-led citizen science projects; ‘producing knowledge’ around the processes and impacts of citizen science approaches; ‘fostering networks’ for ongoing collaboration and support; and ‘brokering new and existing research’ on citizen science to policy and practice stakeholders. An overview of the core project components, and how these relate to the knowledge mobilisation domains are provided in Table 2 and described in more detail below.

**Table 2:** Overview of knowledge mobilisation domains and their relationship to project components

	Overview	Stakeholder-led projects	Resource development	Community of Practice (CoP)	Outreach
Researching in practice	Researching in practice refers to research and implementation occurring simultaneously, often through co-produced research which brings together stakeholders and emphasises learning-by-doing	X			
Producing knowledge	Knowledge products can include academic publications, research summaries and web portals for disseminating theoretical or empirical knowledge with key stakeholders	X	X		
Brokering knowledge	Brokering knowledge includes activities that seek to share research with stakeholders, both through ‘push’ approaches to research translation and relational models that emphasise knowledge exchange between different stakeholders	X	X	X	X
Fostering networks	Fostering networks involves developing connections and collaborations to shape and share expertise, recognising the socially and contextually situated nature of knowledge and tacit knowledge that stakeholders bring	X		X	X

#### Supporting stakeholder-led citizen science projects

The concept of *researching in practice* is central to our program of work. As shown in Fig. 1, our program of work includes four citizen science projects being led (and resourced) by our project partners. We are working with these partners in an ongoing manner to provide support with the design and implementation of these citizen science projects (*brokering knowledge*), including an embedded developmental evaluation in which we are continually collecting, analysing and sharing data to support ongoing decision making around the use of citizen science (see Rowbotham et al*.* [47] for more detail on our approach to the evaluation of these four projects). Regular meetings with each of the citizen science project teams enables ongoing reflection on progress and challenges and opportunities for collaborative problem solving. To facilitate connections across projects and enable peer-learning, we host quarterly whole-of-project meetings, in which we bring together project partners to discuss progress and challenges related to their individual projects (*fostering networks*). Through these four stakeholder-led projects we will *produce knowledge* about the application of citizen science approaches in policy and practice contexts, including publications and other outputs reporting on the insights gained through the developmental evaluation in order to build the knowledge base on citizen science.

#### Developing citizen science resources

The creation and dissemination of “research-based knowledge ‘products’” [48] is an important element of knowledge mobilisation. As highlighted earlier, a priority for our policy and practice stakeholders was the need for evidence and examples to demonstrate the feasibility of citizen science approaches, and resources to support the application of citizen science approaches in prevention. In addition to academic publications, we are developing a set of resources to address information and support needs related to the application of citizen science approaches (*producing knowledge*). We have so far produced: a fact sheet introducing citizen science approaches and highlighting examples from the literature; a short, animated explainer video to introduce the key principles of citizen science; and a series of case studies to share reflections and insights on the use of citizen science approaches within the projects being led by our partners. Additional planned resources include fact sheets to assist in deciding when to use citizen science approaches and tools and technologies for citizen science, and evidence briefs of academic outputs from the program of work. We are also developing a workshop to introduce stakeholders to citizen science approaches. Throughout this process, we are working closely with project partners to make sure that the knowledge products developed are relevant to their needs (*knowledge brokering*). This includes gathering feedback on resources during the development process and co-authorship of academic outputs. All of our resources are hosted on The Australian Prevention Partnership Centre (TAPPC) website [42], and wherever possible academic outputs will be published in open-access format to enable stakeholders to easily access and share these.

#### Community of Practice for citizen science in prevention

*Fostering networks* is crucial across several elements of our program of work and includes providing opportunities for developing connections between project partners and building a wider network of stakeholders interested in citizen science in prevention. A key component of our approach to *fostering networks* is the establishment of a citizen science CoP to bring together a wider group of stakeholders working across academic, policy, practice and community settings who have an interest in these approaches. The objective of the CoP is to provide a space for stakeholders to: share insights, learnings and experiences; build capacity for collaboration between communities, researchers and policy and practice stakeholders; provide assistance with design, implementation and evaluation challenges; develop best practice resources and strategies; and offer mutual support. We worked closely with project partners to develop the CoP, including a series of informal discussions followed by a CoP planning workshop at the end of 2020 (*brokering knowledge*). Sessions are held bimonthly and alternate between a seminar format delivered by invited speakers with experience in the use of citizen science, and workshop sessions to explore topics related to citizen science in prevention. Sessions have focused on recruitment and engagement of citizen scientists, designing and implementing citizen science projects, and generating awareness and acceptance of citizen science approaches. To enable us to capture and share insights from these sessions we have also engaged a graphic illustrator who produces visual summaries of the CoP sessions [42].

#### Outreach activities

The final component of our capacity building approach involves citizen science presentations and workshops to a broader network of government and non-government organisations and groups (*fostering networks*), including local health districts and public health agencies, and discussions with individuals and project teams seeking advice and support in the application of citizen science approaches (*knowledge brokering*). Through these activities we have established new collaborations to support the development and implementation of new citizen science projects by local health districts and academic researchers.

### Phase 2: Evaluating our approach to building capacity in citizen science

To inform future capacity building efforts, we have developed a comprehensive evaluation framework, drawing on the community knowledge mobilisation framework [49] and other relevant literature [50–52]. Within the evaluation we aim to determine the extent of policy and practice stakeholder engagement with our capacity building activities (*reach*), whether they found them useful and relevant to their work (*satisfaction*) and whether there had been any changes in knowledge, attitudes, skills, practices, networks, or decisions as a result of engagement with the capacity building activities (*impacts*). This mixed methods evaluation will draw on a range of existing project data, including interviews conducted as part of the developmental evaluation of the four stakeholder-led citizen science projects (see Rowbotham, Laird [47] for more detail), CoP post-session feedback surveys, and project records and online metadata (e.g. website traffic, newsletter views). In addition, we will administer an evaluation survey at the end of the CSP project to provide an opportunity for policy and practice stakeholders who have engaged with any component of our capacity building work to provide feedback on the level of participation, utilisation, satisfaction and impacts of capacity building activities, in addition to any suggestions for future capacity building work. All stakeholders known to have engaged with our capacity building activities, including partners, implementers and other stakeholders who have been recruited as part of our developmental evaluation [47] will be invited to participate in this survey.

Table 3 outlines the evaluation questions, indicators, and data collection methods across our evaluation domains.

**Table 3:** Evaluation questions, indicators, and data collection methods

Evaluation domains and questions	Indicators	Data collection methods
Reach: To what extent were policy and practice stakeholders engaged with capacity building activities?	CoP membership numbers Attendance at project meetings, CoP sessions and outreach presentations and online views of recorded sessions Views of online resources and newsletters Number and type of new partnerships developed	Project evaluation survey Project records and online metadata
Satisfaction: To what extent were policy and practice stakeholders satisfied with capacity building activities (including relevance and usefulness of activities)?	Satisfaction with capacity building activities and perceived relevance to practice Perceived utility of outputs and knowledge gained	Project evaluation survey Interviews CoP post session surveys
Impact: What were the impacts of this program of work (i.e., were any changes attributable to this program of work)?	Instrumental changes (e.g., to plans, decisions, behaviours, practices, actions, policies) Conceptual changes (e.g., to knowledge, attitudes, awareness) Capability-building changes (e.g., to skills and expertise) Enduring connectivity changes (e.g., to number and quality of relationships and trust)	Project evaluation survey Interviews CoP post session surveys

The evaluation has been approved by the University of Sydney Human Research Ethics Committee (Ref 2022/620 and 2022/647).

## Discussion

Citizen science approaches in prevention can support the generation of more needs-focused and policy and practice relevant evidence. In addition, the use of citizen science approaches has the potential to increase public understanding of, and acceptance for policies and programs aimed at improving health and wellbeing, and can provide policymakers and practitioners in health promotion with a means of accessing locally relevant evidence to inform actions. This paper has outlined a knowledge mobilisation approach to building capacity in the use of these approaches through support, reflection and learning from developmental evaluation findings, and establishment of supportive networks and resources.

To our knowledge, this is the first initiative aimed at building capacity in the application of citizen science approaches in health promotion and public health. By drawing on the principles of knowledge mobilisation we have developed a range of strategies to build capacity including gaining hands-on project experience (*researching in practice*); sharing advice, support and evidence to support the use of citizen science (*brokering knowledge*); contributing to the evidence base on the feasibility, acceptability and impacts of citizen science in public health (*producing knowledge*); and bringing together stakeholders to share challenges, experiences and insights (*fostering network*s). This has enabled us to co-design a multifaceted program of work that is responsive to the capacity building needs of policymakers and practitioners in health promotion.

Central to applying this knowledge mobilisation approach is working in partnership with relevant stakeholders. As argued by Moss [53] “knowledge mobilisation is not just about moving a clearly defined set of ideas, concepts, research techniques or information from here to there. Rather it is about grappling with which forms of knowledge are apt in which contexts and how they can be strengthened through use”. Phipps and Shapson [54] highlight the need for more collaboration between researchers and knowledge users (in this case health promotion practitioners and policymakers) for knowledge mobilisation, which encompasses methods of knowledge co-production, exchange, transfer and translation. Our partnership with health promotion policymakers and practitioners is proving critical to the co-production of knowledge concerning the development, implementation, and impacts of these approaches in practice, and to informing capacity building needs so that support and resources are tailored accordingly.

Another aspect that is key to this approach to capacity building is the use of interactive and participatory approaches to dissemination. To date there has been a lag in the application of relational approaches to knowledge mobilisation by research agencies, such as the establishment of networks or holding of events to enable knowledge exchange, rather than traditional ‘push’ and ‘pull’ approaches involving the production of knowledge outputs such as evidence summaries and research papers [55]. The co-produced nature of this work and the establishment of the CoP and other opportunities to bring stakeholders together allows for interaction between researchers and knowledge users across all capacity building activities.

The CSP project commenced mid-2020 and through ongoing discussions with our project partners and other stakeholders we have been able to adapt and improve the program of work, ensuring it is aligned with stakeholder needs, particularly as their own knowledge and skills in citizen science develop. For example, some of the emerging challenges encountered by project partners have included identification of the most appropriate methods to gather data using a citizen science approach, completing applications for ethical approval, and strategies to engage and support citizen scientists in their projects. By working closely with project partners through the development and implementation of their citizen science projects we are able to tailor capacity building efforts to support the emerging needs of project partners. Early feedback via informal discussions and post-event surveys has endorsed the relevance and utility of the activities and resources provided and the opportunities for stakeholders to come together to share insights. Our outreach activities, including discussions and presentations for various external stakeholders have also resulted in several new collaborations to support the development of citizen science projects with other agencies, further indicating the success of our capacity building efforts. The next step for this project will be to undertake a more formal, in-depth evaluation as outlined in this paper.

Whilst conceptually promising, we acknowledge that the complexity of a multifaceted partnership approach may also present some challenges. The first reflects the need to consider engagement strategies for policy and practice stakeholders as their paid roles may not allow for the desired engagement in capacity building activities [30]. Engagement has been facilitated by the move towards online events since COVID-19, which increases access for interstate stakeholders. This project is also working to ensure activities are recorded or summarised and made available online. The other consideration is the widely acknowledged challenges in evaluating knowledge mobilisation approaches [48]. The evaluation of this program of work has been adapted over time to measure impacts at the individual and organisational levels in relation to capacity to use citizen science methods in policy and practice contexts.

Embedding citizen science approaches has the potential to strengthen community engagement in all aspects of public health and chronic disease prevention policy and practice, from priority setting to design and evaluation of initiatives. This paper outlines the first known approach to support policy and practice stakeholders to apply and sustain the use of citizen science approaches in health promotion. It demonstrates how knowledge mobilisation principles and methods can be applied in a collaborative and multifaceted program of work to facilitate the use citizen science approaches in public health, with potential applications in other fields of policy and practice.

## Data Availability

Not applicable.

## Abbreviations

CoP: Community of Practice
CSP: Citizen Science in Prevention project

## Footnotes

1 ‘Citizen science’ is the widely recognised and accepted terminology used to refer to the range of activities that actively involve the public in science. However, this term is not unproblematic, particularly as the term ‘citizen’ may be understood by some to refer to national citizenship, which can be a sensitive issue. In line with others in the discipline, within this paper “citizen” is equivalent to the concept of the “global citizen”, which includes any person interested in participating in citizen science.
